# Cell morphology governs directional control in swimming bacteria

**DOI:** 10.1038/s41598-017-01565-y

**Published:** 2017-05-17

**Authors:** Òscar Guadayol, Katie L. Thornton, Stuart Humphries

**Affiliations:** 1 School of Life Sciences, University of Lincoln, Joseph Banks Laboratories, Green Lane, Lincoln LN6 7DL UK; 2 Department of Physics, University of York, Heslington, York, YO10 5DD UK

## Abstract

The ability to rapidly detect and track nutrient gradients is key to the ecological success of motile bacteria in aquatic systems. Consequently, bacteria have evolved a number of chemotactic strategies that consist of sequences of straight runs and reorientations. Theoretically, both phases are affected by fluid drag and Brownian motion, which are themselves governed by cell geometry. Here, we experimentally explore the effect of cell length on control of swimming direction. We subjected *Escherichia coli* to an antibiotic to obtain motile cells of different lengths, and characterized their swimming patterns in a homogeneous medium. As cells elongated, angles between runs became smaller, forcing a change from a run-and-tumble to a run-and-stop/reverse pattern. Our results show that changes in the motility pattern of microorganisms can be induced by simple morphological variation, and raise the possibility that changes in swimming pattern may be triggered by both morphological plasticity and selection on morphology.

## Introduction

Bacteria swimming in aquatic systems must navigate a diluted chemical landscape, where the pressure for efficiently locating and tracking nutrient gradients is high (e.g. ref. 1). In response to this pressure, bacteria have evolved a number of chemotactic strategies that allow them to sense and direct their movement towards nutrient sources. Several such strategies have been described, all of them consisting of a sequence of run phases, in which the cell swims in an approximately straight line, interspersed with reorientation phases, which can be active tumbles^2^, arcs^3^, stops^4^ or reversals^5, 6^. By adjusting both the relative frequency and length of these phases (i.e., by biasing the random walk described by their trajectory), as well as their swimming speed^7, 8^, cells are able to adapt to the changing local chemical environment and successfully track nutrient gradients.

In order to efficiently perform these chemotactic strategies, control of direction is crucial. As they run, bacteria detect changes in environmental chemical cues through a complex pathway of signalling proteins^9^. Cells respond to these changes either by readjusting their direction with a reorientation event or by prolonging the run. Since these changes are most commonly detected temporally throughout a run^10^, it is critical that cells maintain straight trajectories during runs in order to obtain meaningful information and adapt their behaviour accordingly. However, because of their small size, the ability of bacteria to swim straight and to change direction depends on the resistance of the cell to being rotated (viscous resistance), which is parameterized by the rotational friction coefficient (*f*_*r*_). During runs, rotational Brownian motion, which is inversely related to *f*_*r*_, causes the cell to gradually lose its orientation and so effectively limits the length of the run. During reorientations, *f*_*r*_ imposes a limit to the amplitude of the turn achievable.

The rotational friction coefficient *f*_*r*_ is dependent on the size^11, 12^ and shape of the cell^13^. Several predictions have been made on the basis of models assuming both spherical^7, 12, 14^ and ellipsoid of revolution^13^ shaped cells, particularly regarding the length of runs. For example, assuming a spherical cell Berg and Brown^2^ predicted a loss in orientation of about 30° per second for an *Escherichia coli* cell running in a medium of high viscosity (*η* = 2.7 kg m^−1^s^−1^), which was similar to observed deviations from a straight path. Dusenbery^10, 13^ predicted that long prolate ellipsoids are potentially best at detecting nutrient gradients because they can run straight for longer periods of time. Mitchell^7, 12^ hypothesized that flagella can stabilize run trajectories against rotational diffusion by effectively increasing the length of the cell. Finally, Locsei and Pedley^15^ suggested that the loss of directionality during a run in *E. coli* is higher than expected from Brownian motion alone due to the wobbly swimming caused by inefficient bundling of flagella. Nevertheless, despite a growing number of theoretical predictions and the increased awareness of the high morphological variability in bacteria and of its importance (e.g. refs 16 and 17), there is little empirical evidence of the effect that size, and particularly shape (defined as a quantitative geometrical parameter rather than a categorical one), have on the directionality of swimming bacteria. The aim of the present study was to experimentally validate our current theoretical understanding of how cell aspect ratio influences both the length of the runs and the amplitude of reorientation events.

We treated a chemotactic strain of *E. coli* with cephalexin to obtain a range of motile cells of different aspect ratios (that is, ratios between the longest and the shortest dimension). Cephalexin is a β-lactam antibiotic that increases the length of *E. coli* by binding with FtsI (also called PBP3), one of the proteins involved in the septal ring formation during division^18^. Thus, cephalexin stops cells from dividing without further altering their growth rate, physiology or flagellar motility^19–21^. We tested the predictions of the ellipsoid model for *f*_*r*_ and for Brownian diffusivity (inversely related to *f*_*r*_). Specifically, we assessed the effects of an increase in cell length on the ability of bacteria to both tumble at a random angle and to run straight. Our results show a tight relationship between the aspect ratio of the cell and its ability to change direction during a reorientation event. For example, tumble angles (that is, angles between consecutive runs) in cells approaching an aspect ratio of one (spherical cells) were distributed uniformly, whereas elongated cells showed restricted tumble angles. These changes in tumble angle led to a change in the swimming mode, from a run-and-tumble pattern to a run-and-stop/reverse one. We also show how the ability of a cell to maintain straight runs increases with cell length.

## Results

### Cell geometry

In order to characterize swimming behaviour of motile cells of different lengths in the absence of chemical gradients, a chemotactic strain of *E. coli* (AW405) was grown in two flasks of minimal media^21^. Cephalexin was added in one of the flasks to induce cell elongation and both treatments were monitored over time for changes in cell morphology and swimming behaviour using phase-contrast microscopy (see Methods for a detailed description of experimental design). The average cell width of *E*. *coli* cells was 0.7 ± 0.1 µm (mean ± s.d.). The mean length of the control untreated population in minimal media was 1.7 ± 0.7 µm. Accordingly, we defined a normal-size class as cells of length 1.7 ± 0.1 µm and of width 0.7 ± 0.1 µm. No significant trend was observed in cell length in the control population throughout the experiment (least-squares linear regression model slope) [*b* ± s.e. = −0.1 ± 0.0 µm hour^−1^; *t*(5) = −2.10, *P* = 0.090]. In the cephalexin treatment, two hours after addition cells started to noticeably elongate and reached a maximum average length of 23 ± 19 µm after five hours. The range of average aspect ratios achieved was 2 to 32.

Flagella appeared to be randomly distributed along the cell in both control and treated populations, and the number of flagella increased with cell length (Fig. S1a). Thus, the predicted number of flagella for normal-size cells was 2.8 ± 0.4 (±95% C.I., Fig. S1a), which is similar to previously reported values^22^. Flagella were on average 6.0 ± 2.5 µm long, and the amplitude and pitch of the helical coil were 0.20 ± 0.04 µm and 2.34 ± 0.25 µm (Fig. S1b,c,d), which are comparable to published values^23^.

### Cell tracking and characterization of runs and tumbles

Using phase microscopy, we video-recorded hourly for six hours the increasingly long cells swimming in a homogeneous isotropic medium, and compared their swimming patterns with the control population of untreated, normal-size cells. We recorded a minimum of 540 and a maximum of 2922 swimming cell tracks equal or longer than two seconds for each treatment and time. Tracks were 4.4 seconds long on average in both control and cephalexin treatment, and no apparent trend in mean track length was observed throughout the experiment [*b* ± s.e. = 0.1 ± 0.1 s hour^−1^; *t*(5) = 1.06, *P* = 0.34]. The percentage of cells swimming at the beginning of the experiment was on average ~48%. In the control treatment the percentage of cells swimming progressively decreased to a minimum of 16% after six hours. On the other hand, in the cephalexin treatment the percentage of cells swimming remained high, between 39% and 64%. Thus, there was a systematic difference between both treatments, which could be directly attributed to cephalexin or alternatively could be caused by physiological or chemical differences in the two cultures, which we did not monitor. Whatever the case, when considering only the motile cells we did not find any significant trends in run times, tumble times or tumble angles in the control population, and only very small changes in swimming speed (see below). Only motile cells are used in subsequent analyses.

*E. coli* shows a well-known run-and-tumble behaviour that is controlled by the direction of rotation of the flagella. When all flagella on a cell rotate counter-clockwise (CCW), they form a single bundle and the cell runs, whereas when one or several of them rotate clockwise (CW), this bundle is disentangled and the cell tumbles. Numerous algorithms have been developed to characterize runs and tumbles in bacterial tracks (e.g. refs 24 and 25). However, none of these was readily usable in our dataset because no single threshold speed or angle can be expected to apply to cells of different size and shape. To address this challenge, we used a simple and novel approach that takes into account the probability of a linear displacement or rotation being caused by Brownian motion. This approach does not rely on subjective estimates of thresholds, but is instead grounded in a well-known and tested physical model for prolate ellipsoids of revolution^26^ (see Methods). It takes advantage of two facts. The first is that bacteria operate at very low Reynolds numbers, and therefore inertial forces play a very small role in their swimming. In other words, when cells stop or start moving they do so virtually instantaneously^14^. The second is that *E. coli* cells tumble actively, which means that flagella rotating CW are actually exerting a torque on the cell that rotates it about its minor axes. These facts imply that any translation or rotation that reasonably exceeds the expectations from Brownian motion alone must theoretically be caused by flagellar movement.

Thus, in order to characterize runs and tumbles, we modelled our cells as prolate ellipsoids of revolution, and calculated speed and angular velocity thresholds as the values that had a probability of <0.01 of being caused by Brownian motion. We recorded as a run any portion of an individual track in which speed was above the speed threshold and in which angular velocity was below the angular velocity threshold, with any other portions of the track recorded as tumbles. Examples of tracks in control and cephalexin treated populations are shown in Videos S1, S2 and S3, and in Fig. S2.

#### Swimming behaviour of normal-size bacteria

**Figure 1:**
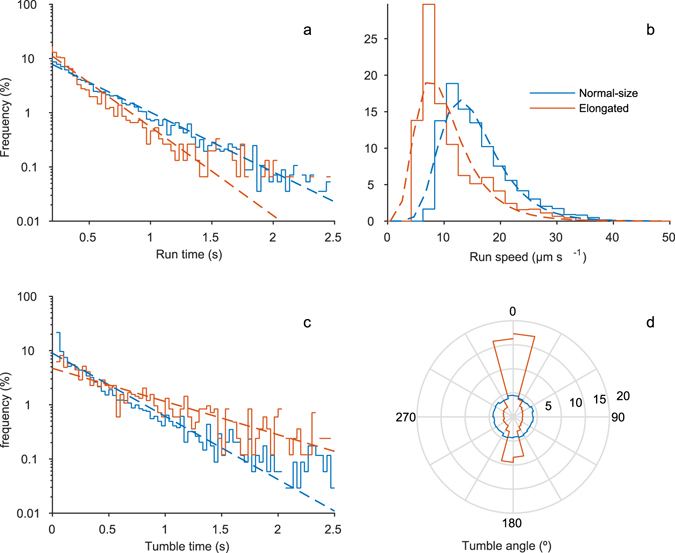
Frequency distributions of (**a**) run times; (**b**) run speed; (**c**) tumble times; and (**d**) tumble angles, for normal-size bacteria (length = 1.7 ± 0.1 µm, in blue) and an example subset of elongated bacteria (length = 10 ± 1 µm, in red). Dashed lines in **a** and **c** are the maximum likelihood estimations of exponential distributions. Dashed lines in **b** are the maximum likelihood estimations of lognormal distributions. Distributions are built from 5687 runs and 4156 tumbles from normal-size cells, and 1521 runs and 970 tumbles from elongated cells.

Both run and tumble times, defined as the duration of runs and tumbles respectively, were approximately exponentially distributed (Fig. 1a,c), as previously observed^2^. Normal-size cells had run times of 0.6 ± 0.4 s (mean ± s.d.), without significant change throughout the experiment [*b* ± *s*.e. = −0.0 ± 0.0 s hour^−1^; *t*(*5*) = −0.50, *P* = 0.641], and run speeds of 16.1 ± 6.1 µm·s^−1^. Average run speed decreased slightly during the experiment in the control treatment [*b* ± *s*.e. = −0.5 ± 0.2 µm s^−1^ hour^−1^; t(5) = −2.72, *P* = 0.042].

The tumble time of normal-size cells was 0.4 ± 0.6 seconds, without a significant trend throughout the experiment [*b* ± s.e. = −0.0 ± 0.0 s hour^−1^; *t*(5) = 1.84, *P* = 0.125]. For tumble angles, normal-size bacteria in the control treatment showed a mean cosine of the tumble angle very close to zero (0.0 ± 0.7), without any significant trend throughout the experiment [*b* ± s.e. = 0.0 ± 0.0 hour^−1^; *t*(5) = 2.04; *P* = 0.097]. This is the expected value when angles are uniformly distributed, and it translates into a mean change in angle between runs of 90 ± 47°.

A compilation of motility parameters reported in the literature for chemotactic wild-type *E. coli* strains free-swimming in homogeneous media is shown in Table S1. Our estimates of run speed and time, and of bacterial diffusivity for normal-size bacteria are within the range of published values, whereas estimates of tumble times and angles are not. Tumble times for normal-size bacteria are longer than in previous studies, and our estimate for the mean tumble angle in normal-size bacteria approaches the value expected in a random distribution (90°, Fig. 1d). This is the first time such a distribution has been reported for chemotactic *E. coli*, for which the distribution is generally skewed towards the forward direction (i.e., tumble angle <90°, Table S1). Such discrepancy may simply be a consequence of the relatively long tumble times we observed. In fact, a very conservative estimate of tumble angular speed, calculated as mean tumble angle divided by mean tumble time, yields the lowest values in Table S1.

An alternative explanation for the discrepancy between the mean tumble angles in our normal-size cells and published values may lay in the different viscosities of the media used in some of the studies. In order to decrease the wobbling of cells and the effect of Brownian motion while swimming^27^, Berg and Brown^2^ added a polymer (hydroxypropyl methylcellulose) that increased the dynamic viscosity of the media to *η* = 2.7·10^3^ kg·m^−1^s^−1^, a value more than three times higher than in normal culture conditions *(η~0.7*6·10^3^ kg·m^−1^ s^−1^). Since the rotational friction coefficient is directly proportional to the viscosity *(η*) of the media^13^, more power is required to turn the cell, and thus the distribution of tumble angles is expected to be skewed towards small angles. To illustrate this, consider two ideal prolate ellipsoidal bacteria of 2 µm in length and 1 µm in width, one swimming in viscous medium as in Berg and Brown, the other in a normal viscosity medium, as in our experiment. The power *(P*_*r*_) needed to rotate a particle is *P*_*r*_ = *Mω*, where *M* is the torque applied to the particle and *ω* is its angular velocity. At low Reynolds numbers, *M* = *f*_*r*_ *ω* ^14^, and thus:

1 $${P}_{r}={f}_{r}{\omega }^{{\rm{2}}}$$

Since frictional coefficients are proportional to viscosity, if we assume that the same power is devoted to turn the two cells, then *η*_*H*_ *ω*_*H*_^*2*^ = *η*_*L*_ *ω*_*L*_^2^, where the subindices *H* and *L* represent high and low viscosity respectively. Solving for *ω*_*L*_, we find that the power required by a bacterium at high viscosity to make an average turn of 68° in 0.14 seconds, such as that observed by Berg and Brown^2^, would produce a 129° turn in a bacterium swimming in normal viscosity medium. This argument does not take into account the effect of increased viscosity on flagellar dynamics^28^. However, it is consistent with reports of decreasing tumble angles with increasing viscosities^29^. Coupled with the idea that *E. coli* cells can switch the direction of runs (that is, can make the end pole become the leading pole)^30^, this suggests that a uniform tumble angle distribution is theoretically feasible. We note, however, that other studies^22, 31, 32^ have reported a forward bias similar to that in ref. 2 with no addition of thickening polymers. Furthermore, Darnton *et al*.^23^, citing their own unpublished data, claimed that increasing viscosity did not alter run-and-tumble statistics. At the very least, the differences in swimming parameters estimates in the literature highlight the sensitivity of these parameters to different experimental conditions and methodologies.

### Run speed vs. aspect ratio

**Figure 2:**
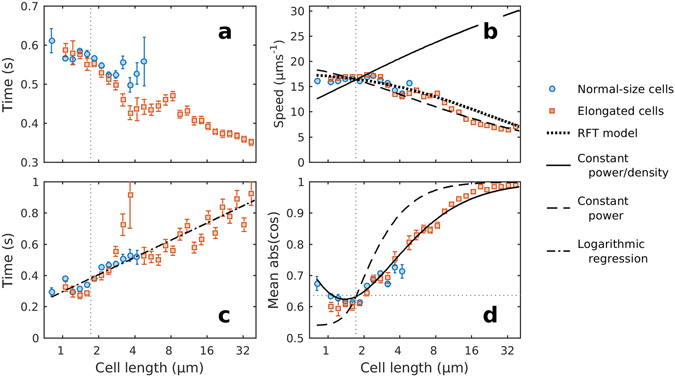
Swimming parameters *vs*. cell length. Run times (**a**), mean run speeds (**b**), tumble times (**c**), and absolute values of the cosines of tumble angles (**d**) for all tracks in each treatment were binned into logarithmically distributed size classes, and then mean values were obtained for each class. Blue circles represent control cells, and red squares represent cephalexin treated cells. Error bars represent the standard error of the means in each size class. Vertical grey dotted lines in all plots mark the average length of untreated *E. coli*. Horizontal grey dotted line in **d** marks the value corresponding to a uniform distribution of angles. Black lines in **b** and **d** represent the power density models (solid line, equations (6) and (8) respectively), the constant power models (dashed line, equations (7) and (9)) and the RFT model (dotted line, equation (3)). Dash-dot line in **c** is the best least-squares regression model between the logarithm of the tumble angle and the tumble time, after forcing the model to cross the value for normal-size cell: *t* = *t*_*n*_ + *b* log_10_(*l*/*l*_*n*_), where *t* is tumble time, *l* the length of the cell, and subscript *n* refers to normal-size cells estimates [*b* ± s.e. = 0.37 ± 0.02; *R* ^2^ = 0.82; d.f. = 27]. Note the base-2 logarithmic axes for cell length.

In the cephalexin treatment, average run times and speeds decreased to ~0.4 s and 7 µm·s^−1^ respectively for cells longer than 20 µm (Fig. 2a,b). The decrease in swimming speed is consistent with previous studies of cephalexin-treated *E. coli*. Maki *et al*.^21^ reported a speed of 11 ± 2 µm·s^−1^ on 7.5 ± 1.2 µm long cells, while Jaimes-Lizcano *et al*.^33^ found a mean speed of 11.56 ± 5.01 µm·s^−1^ on 27.8 ± 11.9 µm long cells.

To understand this decrease in swimming speed we applied Purcell’s model based on resistive force theory (RFT)^34, 35^, using the average values of geometrical parameters of flagella we estimated from our observations. Briefly, the thrust force (*F*_*thrust*_) generated by a single rigid helical bundle of flagella can be described as^35^:

2 $$-{F}_{thrust}=-{f}_{t}v=Av-B{\omega }_{f},$$

where *f*_*t*_ is the translational frictional drag coefficient along the major axis of the cell, *A* and *B* are elements of the propulsion matrix^34^, *v* is the translational swimming velocity of the cell and *ω*_*f*_ is the angular velocity of the flagellar bundle. Solving for *v* gives:

3 $$v=\frac{B{\omega }_{f}}{{f}_{t}+A}.$$

*A* and *B* can be derived from resistive force theory, and depend on the geometry of the flagellar bundle^35^. We used the observed average length and pitch of flagella to estimate *A* and *B*, and assumed a bundle thickness of 100 nm and a bundle amplitude of 0.25 µm^36, 37^. *f*_*t*_ was estimated using the ellipsoidal model (see Methods), and *ω*_*f*_ was assumed to be 2π × 131 rad/s^35^. While it unrealistically assumes that just one bundle of flagella forms and that the angular velocity of flagella remains constant^38^, this model predicts a decrease in speed as cells elongate that fits the data reasonably well (Fig. 2b), although it lies at slightly higher values.

An alternative way to characterize changes in speed with cell length is to look at the power required to propel the cell. At low Reynolds numbers, the translational power *P*_*t*_ needed to displace and ellipsoid of revolution along its major axis at a speed *v* ^13^:

4 $${P}_{t}={f}_{t}{v}^{{\rm{2}}},$$

Assuming that the elongation induced by cephalexin does not alter the physiology of motile cells, the power exerted by cells of different size when swimming can be expected to lay between two extreme scenarios. In the first scenario, the power density is constant, that is, the metabolic power available to a cell is proportional to its volume *V*, and the proportion of metabolic power invested in locomotion is assumed to be constant^11^, therefore:

5 $$\frac{{P}_{t}}{V}=\frac{{P}_{t,n}}{{V}_{n}},$$

where the subscript *n* refers to the parameters for the normal-size bacteria. Using the power density of the normal-size bacteria, combining equations (4) and (5), and solving for *v* leads to:

6 $$v=\sqrt{\frac{{f}_{t,n}}{{f}_{t}}\frac{V}{{V}_{n}}{v}_{n}^{{\rm{2}}}}$$

This model predicts an increase in speed as cells elongate (Fig. 2b).

In the other extreme scenario, the effective power devoted to swimming does not change with size, that is *P*_*t*_ = *c*. Again, using the power requirement of the normal-size cell yields:

7 $$v=\sqrt{\frac{{f}_{t,n}}{{f}_{t}}{v}_{n}^{2}}$$

This model predicts a decrease in speed as cells elongate that fits the empirical data reasonably well (Fig. 2b).

While these are very simple models, both the constant-power and the power density dependent model do allow us to bound the most extreme scenarios with a very small set of experimental parameters. Thus, a more realistic and complex model incorporating parameters such as body and flagellar rotation rates, and number of flagella and/or bundles, would presumably lay between these two scenarios. Since the data already lies near the most conservative of these models, any added complexity relying on more assumptions seems superfluous. Note, however, that the good fits of both the RFT model (equation (3)) and the constant power model (equation (7)) do not necessarily imply that elongated cells are dissipating the same amount of power as the normal-size cells, but rather indicate that only some of the flagella are rotating and/or efficiently propelling the cell. There are a number of possible explanations: only those flagella close to the poles are effectively bundling; flagella are rotating at a slower rate than in normal-size bacteria; or flagella are desynchronized. Experimental evidence suggests that several bundles are formed as cell elongates, but that flagellar rotation rate can decrease with cell length in cephalexin treated *E. coli*.^38^. Furthermore, flagella have been observed to become increasingly desynchronized as cells elongate^20, 39^.

### Directional control during runs

**Figure 3:**
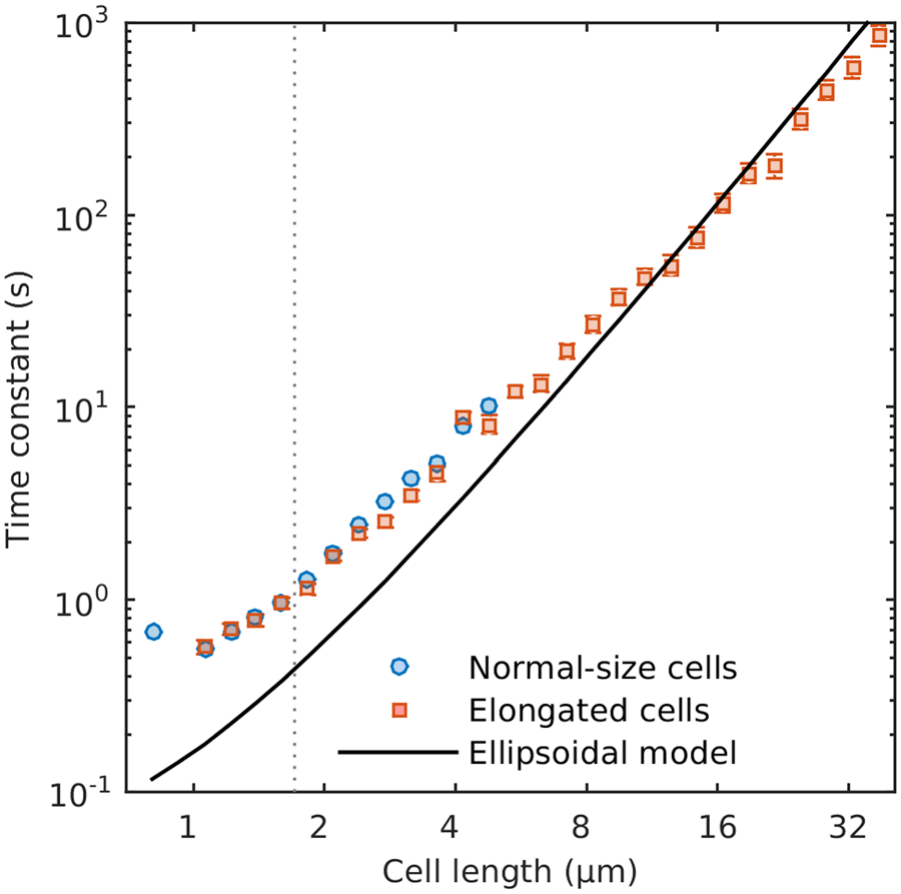
Time constant, i.e. the time it takes for a running cell to lose the original orientation, vs. cell length. Blue circles represent control cells, and red squares represent cephalexin treated cells. Error bars are the 95% confidence intervals of the linear regression least-squares estimate of the time constant (see Methods). Vertical dotted line marks the average length of untreated *E. coli*. Solid line is the theoretical time constant of an ellipsoid of revolution. Note the base-2 logarithmic axis for cell length.

We also observed a large change in the degree of deviation from the initial run orientation as cell length increased. The time constant, that is the time it takes for initial orientation to be lost^10^, increased non-linearly with cell length (Fig. 3). The time constant for normal-size cells was 1.2 seconds, increasing to 41.4 s for cells 10 ± 1 µm long. Thus, the capacity of cells to maintain straight trajectories increased as cells elongated. This response can potentially lead to longer runs, even after considering the decrease in the mean run speed in long cells. For example, given the observed run speeds and time constants, a normal-size cell swimming at ~16 µm·s^−1^ can run for ~19 µm on average before losing its initial orientation. In comparison, a 10 µm long cell swimming at ~11 µm·s^−1^ could run straight for over 450 µm. Thus, changes in cell length can potentially lead to substantially different run lengths.

The time constants are higher than expected from the effect of Brownian motion on ideal ellipsoids (Fig. 3). This is particularly true for the shortest cells. As cells elongate, the empirical values converge with the ellipsoidal model estimates. This supports the idea that flagella can act somewhat as stabilizers of the cell trajectory during runs by effectively increasing the length of the cell^7, 12^, and that this effect is more pronounced on small cells. However, Mitchell’s model of flagellar stabilization leads^7, 12^ to much higher time constants estimates than observed. This is an important point, because maximum run lengths strongly affect chemotactic ability and bacterial diffusivity, and this discrepancy needs to be addressed in further studies.

### Tumble angles vs. aspect ratio

As cells elongated, they spent more time tumbling, but the angles between runs became more restricted, i.e. directional persistence increased with cell length. In the cephalexin treatment tumble times increased non-linearly with cell length to a maximum of 0.9 ± 1.7 seconds for cells between 37 and 40 µm in length (Fig. 2c). For tumble angles, in the cephalexin treatment the mean cosine increased non-linearly with cell length and plateaued at around 0.4 for ~15 µm long cells (Fig. S3). This value corresponds to a mean change in angle of about 69°. In fact, tumbling in long cells was much more restricted than this, but our method does not discriminate between 180° flips and reversals of motion. Since reversals of motion (>90°) result in negative cosines that pull the average towards 90°, to estimate the average turning angle we used the mean of the absolute of the cosine, which is insensitive to the swimming direction and thus best reflects the restriction to turning imposed by shape (Fig. 2d).

Thus, the lengthening of trajectories comes at the expense of the capacity of bacteria to tumble (Fig. 2d). In the shortest cells, changes in angles between runs were approximately uniformly distributed between −π and π. As cells elongated, the distribution became progressively skewed towards small angles. A similar result has been reported for *Vibrio alginolyticus* during flicks^40^. To explain this trend, arguments analogous to those we use for swimming speed can be applied to the angular velocity of the cell body (*ω*). The power used to turn a cell *P*_*r*_ is proportional to the square root of the angular velocity (equation (1)), which we estimated conservatively as the change in angle during a tumble divided by the tumble duration.

As in the case of swimming speed, if we assume no physiological alteration in cephalexin-elongated cells, two extreme scenarios are possible: the power available to generate a turn can increase with cell volume or remain constant. In the first case, the predicted angular velocity for a given cell length is:

8 $$\omega =\sqrt{\frac{{f}_{r,n}}{{f}_{r}}\frac{V}{{V}_{n}}{\omega }_{n}^{{\rm{2}}}},$$

where the subscript *n* refers, as before, to the parameters for the normal-size bacteria. In a constant power scenario, the angular velocity becomes:

9 $$\omega =\sqrt{\frac{{f}_{r,n}}{{f}_{r}}{\omega }_{n}^{{\rm{2}}}}$$

To estimate theoretical values for the maximum tumble angles, we multiplied the measured angular velocities by tumble times obtained from a logarithmic regression between our cell length data and measured tumble times (Fig. 2c). We assumed that tumble angles for a given cell length were randomly distributed between zero and the maximum tumble angles. We then estimated the mean of the absolute value of the cosines of theoretical tumble angles (*α*) as:

10 $$\overline{(\cos (\alpha ))}=\frac{{\rm{1}}}{{\alpha }_{m}}{\int }_{{\rm{0}}}^{{\alpha }_{m}}(\cos (\alpha ))d\alpha $$

where *α*_*m*_ is the maximum tumble angle. The resulting curves are plotted in Fig. 2d alongside the empirical values. As expected, tumble angles become more restricted in the constant power scenario than in the density dependent scenario, as less power is available to turn the cell.

Despite the conservative nature of these theoretical estimates, the data are close to the power density model (Fig. 2d). This trend suggests that the increase in rotational friction associated with an increase in length is effectively restricting the capacity of a cell to tumble. Since *E. coli* cells can run with either pole forwards^30^, this mechanism translates into the back-and-forth movement observed in elongated cells (Fig. 1d, Fig. S2,Video S3)^21^.

### Bacterial diffusivities

**Figure 4:**
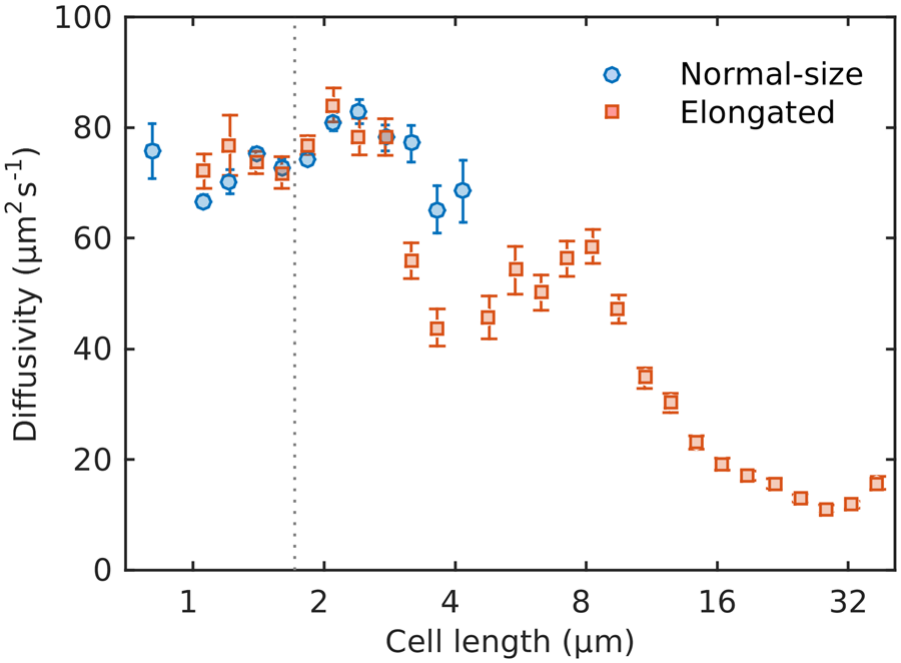
Bacterial diffusivities *vs*. cell length. Blue circles represent control cells, and red squares represent cephalexin treated cells. Vertical dotted line marks the average length of untreated *E. coli*. Error bars are the standard error of the estimates. Note the base-2 logarithmic axis for cell length.

We estimated bacterial diffusivities in two independent ways, one that used run and tumbles statistics^14^ (Fig. 4), and the other based on Taylor’s equation (Fig. S4), for the diffusion of particles in turbulent flows^41^, and which has been applied to model random walks of planktonic organisms^42, 43^. The two estimates of bacterial diffusivities yielded different values. Using the first approach, we obtained a 2-dimensional diffusivity for normal sized cells of 72 ± 1 µm^2^ s^−1^ (±s.e.), whereas Taylor’s approach yielded a value of 131 ± 1 µm^2^ s^−1^. Both values are within the range of published values (Table S1). Nevertheless, the trends observed for both estimates against cell length were similar. First, there is a general tendency for diffusivity to decrease with increasing cell length. Second, there is a local peak in diffusivity around a length of 10 µm, which coincides with local peaks observed in run times and speed (Fig. 2a,b).

## Discussion

The elongation of *E. coli* induced changes in all swimming parameters. Some of these effects (namely on swimming speed and run and tumble lengths) are, as discussed below, likely resulting from desynchronization of flagella, and illustrate the optimality of the shape and size of the wild type *E. coli* to its motility strategy (and viceversa). Other changes (namely on tumble angle and run deviation) were imposed by purely physical constraints. Such changes are not the result of an active behavioural strategy, but rather a physically imposed mechanism.

As cells elongated, runs became shorter and tumbles longer (Fig. 2a,c) and so the tumble bias (the average time a cell spends tumbling) increased with cell length. This pattern is consistent with the “veto model” for run and tumbling behaviour in *E. coli* ^22, 44^. In this model, a cell tumbles whenever any one of its flagella is rotating CW, and is thus breaking the bundle of flagella. The fraction of time a cell spends tumbling depends on the number of flagella and the degree of coupling among them^44^. Both parameters can be expected to change with size and shape. First, the number of flagella increases with cell length (Fig. S1a)^21, 38^. Second, the degree of correlation between flagella is dependent on the cytoplasmic content of the signalling protein CheY in its phosphorylated form (CheY-P), which is coupled to the concentration of chemical stimuli in the environment. The transport of CheY-P across the cytoplasm is driven by molecular diffusion, and thus the time CheY-P takes to diffuse along the cell increases nonlinearly with cell length. For example, given a diffusion rate^45^ of ~10 µm·s^−2^, a CheY-P molecule would diffuse the entire length of normal-size *E. coli* in about 0.15 seconds, but it would take five seconds to diffuse along a 10 µm long cell. Furthermore, CheY-P undergoes dephosphorylation as it diffuses, decreasing in strength by one-third for every 2 µm travelled^39^. The outcome for longer cells is that flagella become increasingly desynchronized, and therefore the probability of a single flagellum rotating CW increases. Following this line of thought, the bundling strategy might only be effective in short rods and could therefore provide a selective pressure to counter the predicted benefits of ever-increasing cell length for swimming speed^10^. In this scenario, membrane potential signalling (e.g. ref. 46) could be more effective at triggering synchronous responses in long peritrichous cells.

The restriction in tumble angles and in run deviation imposed by the change in shape leads to a change in motility pattern, from a run-and-tumble pattern to a run-and-stop/reverse pattern, which is clearly visualized when comparing the tumble distributions of normal size cells and elongated cells (Fig. 1d, Fig. S4). In this new pattern, cell runs are ended by decreases in swimming speed or, when cells are sufficiently long, complete stops. After a stop, cells usually resume running in the same direction as before, but from time to time they reverse direction. Interestingly, similar strategies are found in nature (e.g. ref. 12), and we may therefore assume that they are relatively optimal for specific conditions. In particular, run-and-reverse chemotaxis is a common strategy in marine bacteria^4, 47^, and has also been observed in soil bacteria^48^. In the ocean, this strategy can be advantageous to track stable long one-dimensional gradients^12^, such as those found at the sediment-water interface. Under certain conditions, it may also perform better than other strategies in keeping cells closer to the nutrient source. For example, under moderate turbulent conditions, the interplay between shear and the back-and-forth motility can keep a cell within the phycosphere for longer^49^. It remains to be seen, however, how well this mechanism will work under strong shear with elongated cells that experience longer Jeffery orbit periods^15, 50^. Finally, run-and-reverse motility has also been shown to be potentially advantageous in porous media, such as soils and sediment^51^. Thus, provided that elongated cells in the presence of nutrient gradients can overcome the strong tumble bias, which we believe is an artefact caused by desynchronization of flagella, treating bacteria with β-lactam antibiotics can be a good experimental approach to test a variety of hypotheses about the performance of the two strategies under different chemical and hydrodynamical conditions.

While the run-and-reverse strategy may be optimal in certain conditions, i.e. to swim towards or stay close to a nutrient source (exploitation), it is unclear how effective it will be at finding new gradients (exploration). The decrease in bacterial diffusivities with increasing cell length (Fig. 4), suggests that long cells will be slower dispersing and therefore less efficient at detecting new nutrient sources than normal-size cells. Since bacterial diffusivity is directly proportional to run speed and time (equation (15)), the decrease in diffusivity is mostly driven by changes in these two parameters that are probably a side effect of cell elongation. However, bacterial diffusivity also increases with decreasing tumble angles. Thus, given the plasticity in swimming speed^8^, it is plausible that elongated *E. coli* cells with a run-and-stop/reverse motility can still be competitive in exploring for new nutrient patches in homogeneous conditions.

Although the run-and-stop/reverse pattern we observed in the elongated cells and the run-and-reverse motion described in many marine species appear similar, they are mechanistically different. Run-and-reverse motility has been described in species with one or several flagella at one of the cell poles, and in these species the reversal of motion is achieved by a reversal of rotation of the flagella. In contrast, reversals in *E. coli* are a consequence of the bundle forming randomly at the alternate pole^30^, which combined with the tumbling suppression due to increased drag, leads to the observed changes in motility pattern in elongated cells. However, we hypothesize that such changes can still have ecological relevance, since they were detected not only in the cephalexin treated cells, but also in the longest cells of the control treatment. Thus, small changes in the length (aspect ratio) of the cells that are well within the natural variability of *E. coli*, can effectively lead to a change in chemotactic strategy. A recent study has shown how the diversity in the expression of proteins involved in chemotaxis within the same population of *E. coli* can lead to different responses to different chemical stimuli^52^. Our results suggest another way in which the morphological plasticity in a natural population can allow a variable response. This strategy may not always work for peritrichous species such as *E. coli*, because elongation is accompanied by decreases in run time and speed. However, we hypothesize that this is a feasible mechanism in polar flagellated bacteria with a length range similar to that of *E. coli*, or in species with membrane potential signalling (e.g. ref. 46), where the increase in size will not lead to flagellar desynchronization and slow responses.

The results presented in this study stress the importance of bacterial size and shape in the control of direction, and ultimately, in the efficiency of different chemotactic strategies. This experimental procedure can be used to assess the performance of different chemotactic strategies under different chemical and physical landscapes. Furthermore, our results exemplify how morphological changes, quite within the range of natural populations, can lead to important changes in the motility pattern of microbes.

## Methods

### Growth conditions and media

A single experiment was performed in a homogeneous medium to characterize swimming behaviour in the absence of chemical gradients. A chemotactic strain of *E. coli* AW405 was grown overnight from a glycerol frozen stock in minimal growth medium^21^, at 33 °C and shaken at 200 rpm. The saturated culture was diluted 100-fold into two 125 ml Erlenmeyer flasks with 25 ml of fresh medium and then let grow for about two hours in the same conditions until early exponential growth was achieved (OD_600_~0.2). Cephalexin (Sigma-Aldrich Company Ltd, CAS# 15686-71-2) was then added to one of the flasks to a final concentration of 60 µg·mL^−1^, while the second flask was kept as a control. Reported changes over experimental time in geometrical and swimming parameters (see below) were detected in the control treatment with linear least-squares regression models.

### Video microscopy

Samples of each treatment were taken every hour for 6 hours to characterize swimming performance as cells gradually elongated in the cephalexin treatment. Samples were diluted with minimal medium to OD_600_~0.05 in order to keep a constant concentration of cells throughout the experiment, and then placed in a slide with a cavity of approximately 15–18 mm in diameter and 0.6–0.8 mm in depth (Fisher Scientific UK Ltd, Loughborough, UK). The coverslip was sealed at the edges with silicon grease to avoid any drift in the sample. The slide was placed in a heating plate (PE100-ZAL System, Linkam Scientific Instruments Ltd., Tadworth, UK) and the microscope was enclosed in a purpose-built environmental box, ensuring that the sample was kept at 33 °C during imaging, and that no temperature gradient was established within the sampling volume. Cultures were visualized using a Zeiss AxioVert A1 phase-contrast inverted microscope with a green filter (Carl Zeiss Microscopy GmbH, Jena, Germany). Cells were visualized in a 2-dimensional focal plane in the middle of the cavity, at a minimum distance of 150 µm from any wall, which is sufficient to neglect surface effects^25^. For each sampling, four 120 s long videos were recorded at 30 frames per second (fps) using a Hamamatsu ORCA-Flash2.8 camera (Hamamatsu Photonics K.K., Hamamatsu, Japan), a condenser with a numerical aperture of 0.55 and a 10X LD objective with a numerical aperture of 0.25. The resulting lateral resolution is 2.75 pixels per µm and the depth of field was 12.4 µm. Frames were 696 by 696 8-bit grayscale pixels in size, giving fields of view of 242 by 242 µm. Videos were captured using HCImage Live software.

### Particle detection and characterization

All image analyses were performed using in-house written Matlab code, available online^53^. A background image was composed as the median of all frames, and then subtracted from each frame to remove noise. A median filter with a window the size of the area of a particle of 1 µm in diameter was applied to each frame to further reduce noise. Particles were then detected heuristically as regions in each frame in which pixel intensity was above a visually determined threshold. Light conditions, frame rate and shutter speed were kept constant throughout the experiment to avoid inconsistencies in the intensity thresholding. Particles with an area smaller than that of a 1 µm spherical cell were discarded. Centroids of each particle in each frame were estimated using function *regionprops* from the Image Processing Matlab toolbox. Tracks were built from overlapping particles in consecutive frames^24^.

Cells length and width were determined as follows. Particle boundaries in each frame were thinned to lines using *bwmorph* function from the Image Processing Matlab toolbox, and these lines were further extended to reach the boundaries. Cell lengths were calculated as the arc length of these skeleton lines. These estimates were tightly correlated to, although consistently lower than, the major axes of the ellipses that have the same normalized second central moments as the regions, as calculated by function *regionprops*. Because cell widths were comparable to the errors when using 10X magnification objective, they were determined manually from subsamples visualized under a 100X objective using function *imdistline* from the Image Processing Matlab toolbox.

Phase-contrast microscopy is subject to diffraction effects that lead to overestimations of bacterial size^54, 55^. Furthermore, the determination of the pixel intensity threshold depends on the light intensity and can add some uncertainty. Thus, in order to calibrate bacterial dimensions from images obtained with phase-contrast microscopy, we used silica beads of four diameters (0.50 ± 0.013, 0.75 ± 0.03, 0.997 ± 0.026 and 1.55 ± 0.04 µm; Microparticles Gmbh, Berlin, Germany) with a refractive index of 1.42^56^, very close to that of *E. coli* (1.388^57^). We imaged and characterized these beads using the same microscopy settings and image analyses procedures as for the experiment. The smallest beads (0.5 µm) were too small for adequate resolution at the level of magnification used and were therefore discarded. For all other beads, a constant overestimation of 2 pixels (i.e. 0.755 ± 0.03 µm, median ± median absolute deviation) was found, which we subtracted from all our bacterial size measurements.

Orientations of cells (from −π/2 to π/2) between the x-axis and the major axis of the ellipse that had the same second-moments as the particle, were determined using *regionprops*. For short cells (aspect ratio <3), orientations estimated this way were inconsistent, and thus orientations were estimated from the angle change between each triplet of consecutive centroids^25^. The threshold at which orientations estimated from *regionprops* were robust was identified as the aspect ratio at which all run-and-tumble statistics calculated with both methods were not significantly different. The orientation was subsequently modified into a 0–2π range to account for the swimming direction of the particles.

### Tracking

Tracks were built from overlapping particles in consecutive frames^24^. Assuming a minimum size of bacteria of 1.5 µm, and a 30 fps frame rate, any bacteria moving at a speed below 45 µm·s^−1^ would produce tracks. Crossing tracks were split, and tracks shorter than 2 seconds were removed. For each particle, cell length was calculated as the frame-wise median of the estimated lengths of the particle throughout the whole track. Tracks were smoothed with a five frames long moving average (that is with a 0.17 seconds window) in order to minimize uncertainty in the position of centroids. Given a 24 Hz rotation rate of the cell body^23^, this window ensures several (~4) full body rotations are averaged and the uncertainty associated with wobbling is filtered out.

### Ellipsoidal model

The continuous collisions of molecules against a particle floating in a liquid make it translate and rotate in a random pattern called Brownian motion. These movements are characterized by translational (*D*_*t*_) and rotational (*D*_*r*_) Brownian diffusivities:

11 $${D}_{t}=kT/{f}_{t}$$

12 $${D}_{r}=kT/{f}_{r}$$

where *k* is the Boltzmann constant and *T* is temperature in Kelvin. Both *f*_*t*_ and *f*_*r*_ are dependent on the size and shape of the particle and the viscosity of the medium. Following Dusenbery^13^, we modelled our bacteria as rigid prolate ellipsoids of revolution, for which rigorous solutions for friction coefficients exist^26^. This model predicts a non-linear increase of both rotational and friction coefficients with increasing cell lengths. We applied equations 1 to 3 in ref. 13, using cell length and width as major and minor axes respectively. We further assumed that the direction of travel was parallel to the longest axis. Dynamic viscosity of the growth medium at 33 °C was calculated using Matlab function *SW_Viscosity* in the Seawater Thermophysical Properties Library^58^, assuming a salinity of 0.

### Discrimination of motile and non-motile cells

A particle was assumed to be moving purely by Brownian motion when the displacements (*d*) from frame to frame along the major axis of the ellipse were distributed following a normal distribution in which *d* = 0 and var(*d*) ≤ 2*D*_*t*_ *t* ^14, 59^, where *t* was the time between measurements in seconds. The first condition was tested with a *t*-test at a 0.001 level of significance, whereas the second was tested using a Chi-square variance test at a 0.001 level of significance. We estimated the percentage of moving bacteria as the frame-wise mean of motile cells divided by all detected cells in each frame.

We checked the efficiency of this method in the following way. A subsample for each treatment and time was killed and fixed by adding glutaraldehyde to a 1% final concentration and frozen at −80 °C for subsequent analyses. Later, these samples were thawed and imaged in the same way as the live samples to characterize Brownian motion of cells of different shape. The method yielded a ratio of false positives of ~1/300, higher than the 1/1000 that would be expected given the level of significance applied, but still low enough to consider the method sound. All non-motile cells were excluded from subsequent analyses.

### Runs and tumbles

In order to characterize runs and tumbles, we assumed that a swimming cell was always either actively running or actively tumbling, and therefore, that any translation or rotation that could not be explained by Brownian motion alone was a run or a tumble, respectively. Thus, we calculated the speed and angular velocity thresholds as the values with a probability of being caused by Brownian motion <0.01. We recorded as a run any portion of an individual track in which speed was above the speed threshold and below the angular velocity threshold, and as tumbles any other portion of the track.

To estimate these thresholds, we took advantage of the fact that both the positions along an axis, and the orientations of a population of particles subjected to Brownian motion follow a normal distribution with standard deviations *σ*_*t*_ = (2*D*_*t*_ *t*)^1/2^ and *σ*_*r*_ = (2*D*_*r*_ *t*)^1/2^ respectively^14, 59^, where *D*_*t*_ and *D*_*r*_ are the translational and rotational diffusivities. Thus, for any given cell, we determined the speed threshold as the value at which, given the cell length, the probability of an observed displacement along the major axis over a time *t* being caused by translational Brownian motion was <0.01. Similarly, the angle threshold was determined as the value at which the probability of a change in orientation about the minor axis over a time *t* being caused by rotational Brownian motion was <0.01. The observed trends were quite insensitive to the value of *t*, although very small values resulted in noisier trends. A good compromise was using the same window length as for tracking (0.17 s, see above), which is sufficient to filter out the noise caused by cell wobbling. Incomplete runs or tumbles, that is, those at the beginning or end of a track, were discarded from subsequent analyses. Additionally, in order to minimize any bias in tumble angle estimations^60^, tumbles that were not flanked by complete runs were also discarded.

Videos S1, S2, and S3 are examples showing the tracking of the control culture (Video S1) and the cephalexin treated culture two hours (Videos S2, with cells of 8.38 ± 3.05 µm mean length) and three hours (Video S3, with cells of 11.9±7.5 µm mean length) after the start of the experiment.

### Angular deviations of runs

As cells run, they are deviated from a straight path by Brownian motion. In a 2-dimensional framework, the mean-square angular deviations *ф* over a period *t* of a cell running follow^14^:

13 $$\langle {{\Phi }}^{2}\rangle =2{t}{{D}}_{{r}{b}}{,}$$

where *D*_*rb*_ is the rotational diffusivity about one of the minor axes. We estimated *ф(t)* during each run as the difference in orientation over a range of periods *t*. *D*_*r*_ was then estimated from the least-squares fit of <*ф* ^2^> *vs. t*. A span of *t* between 0.03 and 0.33 s was used as this was found to be consistently within the linear range for all cell lengths.

A time constant *τ* is then defined as the relevant time required for the initial orientation to be lost, that is, for the root-mean-square of the angular deviations to become one radian^10^. The time constant *τ*_*−a*_ for loss of orientation along the major axis (*a*) of an ellipsoid of revolution of minor axis (*b*) is:

14 $${\tau }_{-a}=\frac{{\rm{1}}}{{\rm{2}}{D}_{rb}}=\frac{{f}_{rb}}{{\rm{2}}kT},$$

where *f*_*rb*_ is the rotational friction coefficient about the minor axis of the ellipsoid.

### Bacterial diffusivities

Bacterial diffusivities *D*_*b*_ for each size class were calculated in two alternative ways. The first one used the statistics obtained from the runs and tumbles analyses applying^61^:

15 $${D}_{b}=\frac{{v}^{{\rm{2}}}\tau }{n({\rm{1}}-\alpha )},$$

where *v* is the mean speed of runs, *τ* is the mean duration of runs in seconds, *n* = 2 is the number of dimensions and *α* is the mean value of the cosine of the angle between successive runs^14^.

A second estimate which does not use any of the statistics derived from the run and tumble methodology was made by applying Taylor’s equation^41^ following^42, 43^. Taylor’s equation describes how much a particle moving in a random walk pattern will be displaced over time:

16 $$RMSD(t)={[{\rm{2}}{v}^{{\rm{2}}}\rho (t-\rho ({\rm{1}}-{e}^{t/\rho }))]}^{{\rm{1}}/{\rm{2}}}$$

where *RMSD* is the root-mean-square net displacements of particles, *t* is time and *ρ* is the correlation time scale, which is related to the run time *t*_r_ and the mean cosine of the tumble angles α^42^:

17 $$\rho =\frac{{t}_{r}}{({\rm{1}}-\alpha )}$$

The *RMSD*(*t*) of tracks in the horizontal plane were estimated as:

18 $$RMSD(t)={(\frac{1}{N}\sum _{i=1}^{N}({(x(i,t)-x(i,0))}^{2}+{(y(i,t)-y(i,0))}^{2}))}^{1/2}$$

where *x* and *y* refer to the position in the horizontal plane of particle *i* at time *t*. The values for *ρ* and *v* were estimated for each size class by fitting RMSD *vs*. time using function *nlintfit* in Matlab. Bacterial diffusivities were then calculated as^42^:

19 $${{D}}_{{b}}={{v}}^{2}\rho /{n}$$

### Size classes

To analyse the effect of elongation on the different swimming parameters, we categorized cells in size classes and calculated ensemble statistics. Given that cell size was approximately exponentially distributed, to avoid an over-representation of the smallest cells we established logarithmic-size bins. Furthermore, this binning ensured a better resolution at the lower size classes, where observed changes in the studied parameters were steeper. Only size classes with over 100 observations were used for subsequent analyses.

### Flagella visualization and characterization

Cells were stained with succinimidyl ester of Cy3 (GE Healthcare UK Lt, CAS# 146368-16-3) following^22, 62^. Briefly, 1 mL samples of *E*. *coli* cultures were washed 3 times in motility buffer (MB) with 0.002% Tween 20 (Sigma-Aldrich CAS# 9005-64-5), prepared as described in ref. 22, by centrifuging 3 times at 1400xG for 12 minutes and resuspending the pellet on MB. Final wash was performed with MB with a pH = 7.5, and the final pellet was adjusted to a volume of ~250 µL. Dye was prepared by diluting a vial of Cy3 diluted in MB at pH = 7.5. This solution was added to the final suspension of bacteria along with 20 µL of NaHCO_3_ (1 M) to shift the pH to ~7.8. The bacterial suspension was shaken at 100 rpm at 33 °C for 90 minutes. Then the suspension was washed free of dye by centrifugation and resuspension as described above. The final resuspension was done with MB with 0.5% (w/V) D-glucose to allow anaerobic motility in microscopic samples.

To quantify the number of flagella on a range of cells of different length, this protocol was repeated every hour during a cephalexin treatment time course using the same design than for the main experiment. To avoid cell division in the treatment population while visualizing the flagella, cephalexin was added to the washing MB at a concentration of 60 µg·mL^−1^.

After staining, samples were placed in a flat slide and the coverslip was sealed at the edges with silicon grease to avoid any drift in the sample. Cells were observed at room temperature with a Nikon Eclipse 80i epifluorescence microscope with a FITC filter box, and a 100X immersion oil objective. Cells were visualized in a 2-dimensional focal plane. Short videos were recorded at 45fps using a Hamamatsu ORCA-Flash2.8 camera. The number of flagella of each cell was determined individually after careful observation of the moving flagella. Both cells and individual flagella were geometrically characterized in a single frame in which they were clearly parallel to the focal plane, by using standard functions in Matlab Image Processing Toolbox. Amplitude and pitch of individual flagella were estimated by identifying the maximum peak of spectral amplitude after applying a fast fourier transform to the flagella positions in an XY plane.

## Electronic supplementary material

Supplementary Material

SREP-16-29914-T-s02.avi

SREP-16-29914-T-s03.avi

SREP-16-29914-T-s04.avi
