# Supplementary material for: Cell morphology governs directional control in swimming bacteria
Source: Sci Rep. 2017 May 17;7:2061. doi: 10.1038/s41598-017-01565-y (PMC5435708; doi:10.1038/s41598-017-01565-y)
Supplement: Supplementary file 1 — Supplementary Material [file 41598_2017_1565_MOESM1_ESM.pdf]

Supplementary material for “Cell morphology governs directional control in swimming bacteria"

Òscar Guadayol, Katie L. Thornton, Stuart Humphries

Supplementary figures

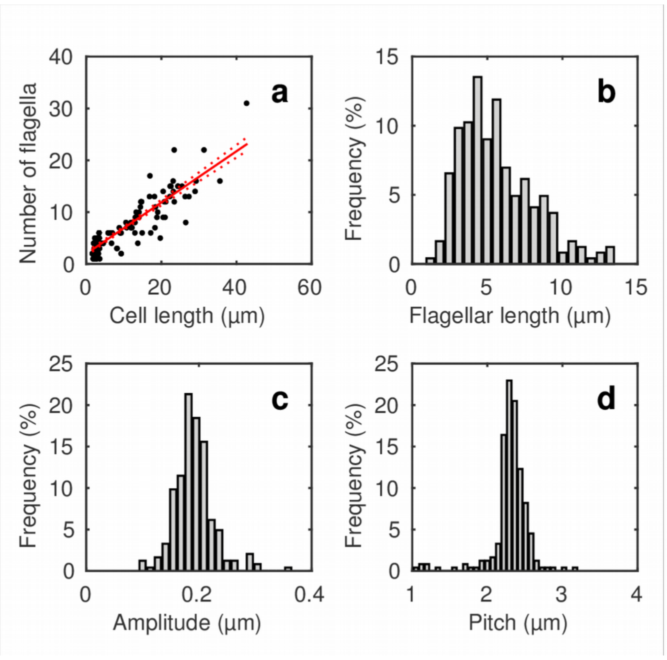

Figure S1: Flagella characterization. **a** shows number of flagella vs cell length. Solid red line shows the best least-squares fit $N_f = 1.97(\pm 0.21) + 0.49(\pm 0.02)\cdot l$ [$R^2 = 0.80$, $F(2, 174) = 715$, $P < 0.001$], where N_f is the number of flagella and l is the cell length in µm. Dashed red lines show the 95% confidence intervals of the model. **b**, **c** and **d** show respectively the frequency distribution of linear length, amplitude and pitch of flagellar filaments.

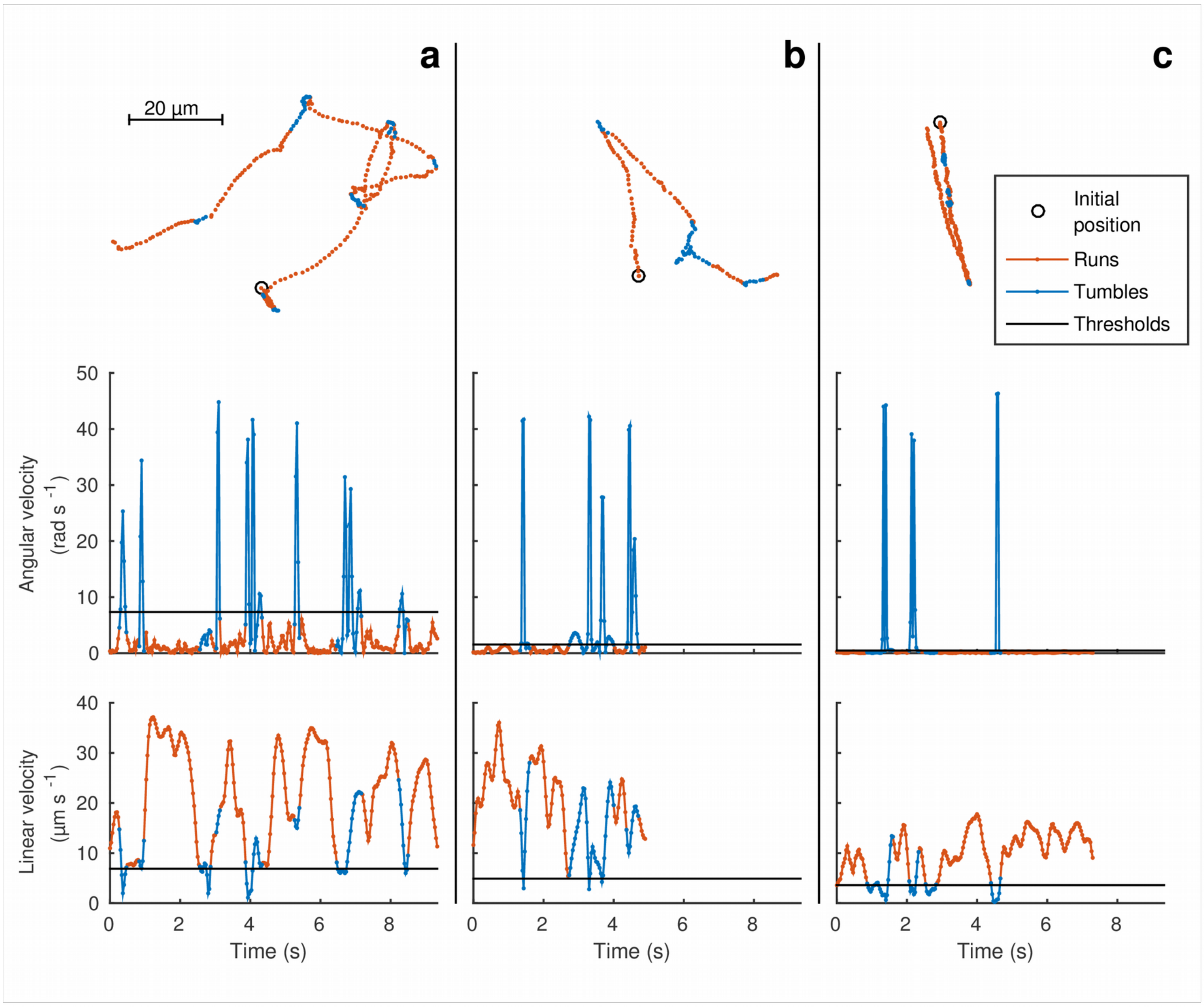

Figure S2: Example tracks for three cells of different cell length. Column **a** corresponds to a cell 1.4µm long, column **b** to a cell 7.8 µm long and column **c** to a cell 19.1 µm long. First row shows the raw tracks; second and third row show the angular and linear velocity, respectively, after applying the moving average filter.

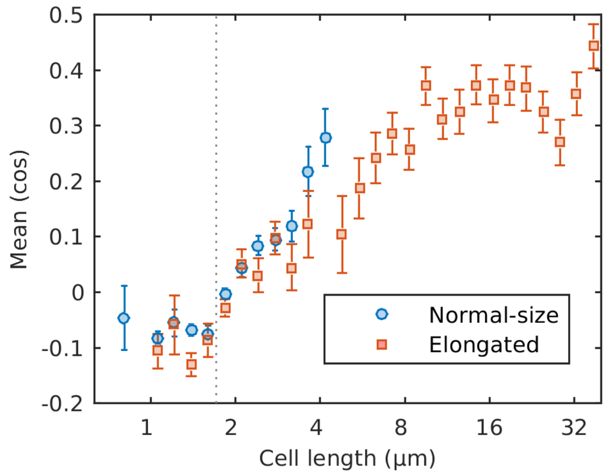

Figure S3: Cosine of cell tumbling angle *vs*. cell length. Blue circles represent control cells, and red squares cephalexin-treated cells. Vertical dotted line marks the average length of untreated *E. coli*. Error bars are the standard errors of the estimates. Note the base-2 logarithmic axis for cell length.

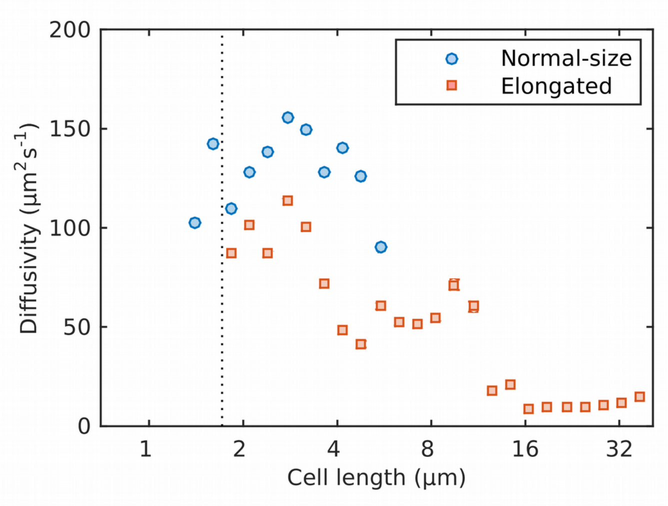

Figure S4: Bacterial diffusivities calculated using Taylor's approach *vs*. cell length. Blue circles represent control cells, and red squares cephalexin-treated cells. Vertical dotted line marks the average length of the untreated *E.* coli. Error bars, which in most cases are smaller than the markers, are the standard errors of the estimates. Note the base-2 logarithmic axis for cell length.

Table S1

Reference	Run speed (μm·s^{-1})	Run time (s)	Tumble angle (deg)	Tumble time (s)	Angular velocity (deg·s^{-1})	Diffusivity (μm^{2}s^{-1})	Strain	Medium
This study	16.1±6.1	0.6±0.4	90±47	0.4±0.6	225±155	72±1*	AW405	Minimal growth medium
[1]	14.2±3.4	0.86±1.18	68.0±36.0	0.14±0.19	485.7	92*	AW405	Motility buffer + methylcellulose
[2]	18.2±7.9		71				RP437	Motility buffer
[3]	-	0.35	~55**	0.19	289	-	RP437	Motility buffer
[4]			58±40	0.14±0.08	414		AW405	Motility buffer + Brij 35
[5]						53.2	RP437	Minimal M9 medium
[6]	18.8±8.2	0.41±0.39	69±1	0.18±0.18	383	75*	RP437	Minimal M9 medium
[7]	14.1±8.0	0.93±1.32	71.3±44.0			~200	AW405	Motility buffer

Table S1. Compilation of literature average values (± s.d.) of run-and-tumble parameters, for wild-type for chemotaxis *E. coli* cells free-swimming in homogeneous isotropic solutions, and away from boundaries. *Calculated from parameters given in original paper using equation (15) in Methods, with reported error being s.e.**Estimated from histogram in Fig. 8C in [25].

References

1. Berg, H. C. & Brown, D. A. Chemotaxis in *Escherichia coli* analysed by three-dimensional tracking. *Nature* **239**, 500–504, doi:10.1038/239500a0 (1972).
2. Deepika, D., Karmakar, R., Tirumkudulu, M. S. & Venkatesh, K. V. Variation in swimming speed of *Escherichia coli* in response to attractant. *Arch. Microbiol.* **197**, 211–222, doi:10.1007/s00203-014-1044-5 (2014).
3. Rosser, G. et al. Novel methods for analysing bacterial tracks reveal persistence in *Rhodobacter sphaeroides*. *PLoS Comput. Biol.* **9**, e1003276, doi:10.1371/journal.pcbi.1003276 (2013).
4. Turner, L., Ryu, W. S. & Berg, H. C. Real-time imaging of fluorescent flagellar filaments. *J. Bacteriol*. **182**, 2793–2801, doi:10.1128/JB.182.10.2793-2801.2000 (2000).
5. Wu, M., Roberts, J. W., Kim, S., Koch, D. L. & DeLisa, M. P. Collective bacterial dynamics revealed using a three-dimensional population-scale defocused particle tracking technique. *Appl. Environ. Microbiol.* **72,** 4987–4994, doi:10.1128/AEM.00158-06 (2006).
6. Saragosti, J. et al. Directional persistence of chemotactic bacteria in a traveling concentration wave. *Proc. Natl. Acad. Sci.* **108**, 16235–16240, doi:10.1073/pnas.1101996108 (2011).
7. Molaei, M., Barry, M., Stocker, R. & Sheng, J. Failed escape: solid surfaces prevent tumbling of *Escherichia coli*. *Phys. Rev. Lett.* **113**, 068103, doi:10.1103/PhysRevLett.113.068103 (2014).

Video legends

Video S1. Example video of the control population two hours after the start of the experiment, with superimposed tracks. Blue tracks correspond to cells not actively moving. Yellow and red tracks correspond to cells actively swimming, where red segments correspond to runs, and yellow segments correspond to tumbles, as characterized by our algorithm.

Video S2. Example video of the cephalexin-treated population two hours after the start of the experiment, when cells were 8.3±3.0 µm in length (average ± standard deviation), with superimposed tracks. Blue tracks correspond to cells not actively moving. Yellow and red tracks correspond to cells actively swimming, where red segments correspond to runs, and yellow segments correspond to tumbles, as characterized by our algorithm.

Video S3. Example video of the cephalexin-treated population three hours after the start of the experiment, when cells were 11.9±7.5 µm in length (average ± standard deviation), with superimposed tracks. Blue tracks correspond to cells not actively moving. Yellow and red tracks correspond to cells actively swimming, where red segments correspond to runs, and yellow segments correspond to tumbles, as characterized by our algorithm.
